# Waste to Wealth Approach: Improved Antimicrobial Properties in Bioactive Hydrogels through Humic Substance–Gelatin Chemical Conjugation

**DOI:** 10.1021/acs.biomac.3c00143

**Published:** 2023-05-11

**Authors:** Virginia Venezia, Mariavittoria Verrillo, Pietro Renato Avallone, Brigida Silvestri, Silvana Cangemi, Rossana Pasquino, Nino Grizzuti, Riccardo Spaccini, Giuseppina Luciani

**Affiliations:** † DICMaPI, Department of Chemical, Materials and Industrial Production Engineering, University of Naples Federico II, Naples 80125, Italy; ‡ DiSt, Department of Structures for Engineering and Architecture, University of Naples Federico II, Naples 80125, Italy; § Department of Agricultural Science, University of Naples Federico II, Portici 80125, Italy; ∥ Department of Civil, Architectural and Environmental Engineering, University of Naples Federico II, Naples 80125, Italy

## Abstract

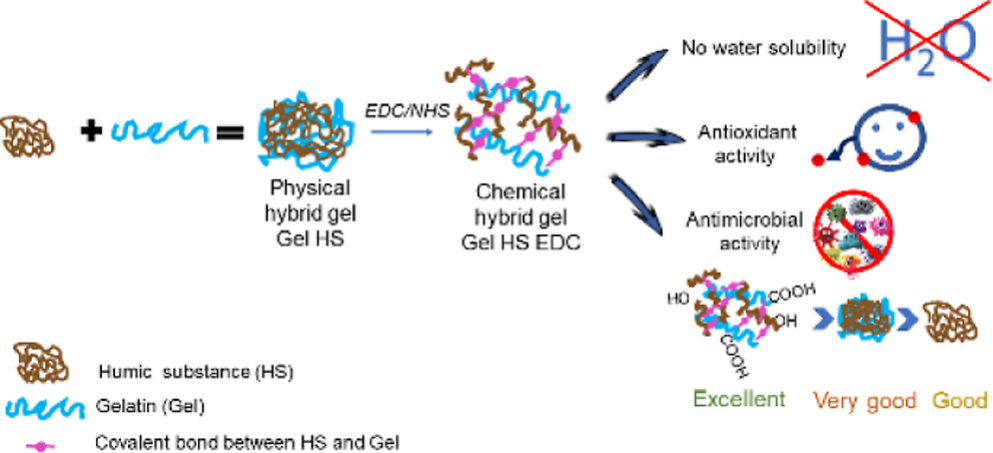

Exploring opportunities for biowaste valorization, herein, humic substances (HS) were combined with gelatin, a hydrophilic biocompatible and bioavailable polymer, to obtain 3D hydrogels. Hybrid gels (Gel HS) were prepared at different HS contents, exploiting physical or chemical cross-linking, through 1-ethyl-(3-3-dimethylaminopropyl)carbodiimide (EDC) chemistry, between HS and gelatin. Physicochemical features were assessed through rheological measurements, X-ray diffraction, attenuated total reflectance (ATR) spectroscopy, nuclear magnetic resonance (NMR) spectroscopy, and scanning electron microscopy (SEM). ATR and NMR spectroscopies suggested the formation of an amide bond between HS and Gel via EDC chemistry. In addition, antioxidant and antimicrobial features toward both Gram(−) and Gram(+) strains were evaluated. HS confers great antioxidant and widespread antibiotic performance to the whole gel. Furthermore, the chemical cross-linking affects the viscoelastic behavior, crystalline structures, water uptake, and functional performance and produces a marked improvement of biocide action.

## Introduction

Biowastes (BWs) hold huge potential to contribute to transition toward a circular model because of their large abundance and remarkable chemical and biological richness. Indeed, industries and academic researchers are spending great efforts to increase the economic and environmental value of BWs by developing strategies for their recycle and conversion into value-added compounds and materials.^1,2^ Among BWs, humic substances (HSs) are the alkali-soluble fraction of natural organic matter that survive the biological and chemical degradation of both vegetal and animal biomasses.^3,4^

They are viewed as a promising and cost-effective source for high-value products and novel biocompatible materials for a wide range of applications spreading from environmental to biomedical field^5^ because of their intriguing properties. Phenolic and carboxylic groups in HSs are primarily responsible for improved plant growth and nutrition,^6−9^ flame retardancy features due to char formation in combustion processes,^10,11^ metal ion chelation, and even redox behavior, providing for remarkable antioxidant, antiviral, and anti-inflammatory activities.^12−14^ Reversible redox chemistry of quinone moieties accounts for both antioxidant and pro-oxidant activities of these bioavailable mixtures because of their ability to generate, stabilize, or scavenge reactive oxygen species (^•^OH, ^•^O_2_^–^, and ^•^OOH), also known as ROS.^15^

Furthermore, the amphiphilic nature of HSs determines self-assembly in an aqueous environment,^16−18^ building up supramolecular structures which can act as metal chelating agents^19,20^ and can interact with organic contaminants.^21−23^ Despite the substantial potential offered by bioavailable HS mixtures, their full technological exploitation is strongly limited by their segregation leakage and/or degradation phenomena in an aqueous environment. Accordingly, they are still mainly considered as waste, and only a small amount is employed for low-value processes including soil amending.

Turning HS incoherent and heterogeneous powders into self-standing and mechanically stable 3D materials could be a promising strategy to scale up HS possible applications. Water is bound to play a key role in the redox equilibria of quinone moieties.^24^ Furthermore, HS supramolecular superstructures make them dynamic systems, which can undergo self-restructuring in water and improve redox activity.^25^

Therefore, the selection of a hydrophilic matrix is envisaged to be the most appropriate solution to upgrade intrinsic HS activity. In this context, 3D porous architectures (hydrogel and aerogel) obtained by gelification with hydrophilic natural organic molecules (i.e., gelatin from porcine skin or chitosan) through physical or chemical interactions stand as the most coherent choice with eco-sustainable goals. Novel bioinspired approaches to the fabrication of biocompatible and biodegradable hydrogel fabrication involved the use of natural polyphenol-like molecules [tannic acid (TA), lignin (LIG), gallic acid (GA), tea polyphenols (TP), etc.^26−28^] that can improve the thermal, mechanical, and functional properties of the final materials.^29^

Gelatin, a natural peptide macromolecule derived from partial hydrolysis of collagen,^30,31^ is one of the most used biopolymers in hydrogel preparations. Its crystalline structure is due to the presence of both ordered triple- and α-helix domains. It is suitable for pharmaceutical, food, or biomedical fields, thanks to its biodegradability, biocompatibility, low cost, widespread availability, and low antigenicity.^32^ Indeed, as a byproduct of meat manufacturing, Food and Drug Administration (FDA) considers it safe.^33,34^ Gelatin’s versatile functionalization offers prospects of effective cross-linking, and its combination with other materials (e.g., metal nanoparticles, carbonaceous, minerals, and polymeric materials exhibiting desired functional properties) allows us to obtain hybrid materials of improved thermomechanical, physicochemical, and functional features.^35^

Indeed, gelatin possesses various functional groups, including aspartic acid −COOH groups, terminal −NH_2_ and −COOH groups, the −NH_2_ group of lysine, the imidazolium group of histidine, and the guanidinium group of arginine, as well as carboxyl and phenolic groups, which can act as potential sites for conjugation opportunities and chemical modifications.

However, high solubility in water of gelatin may be the major disadvantage in terms of tissue engineering, drug encapsulation, and water treatment applications. Therefore, cross-linking methods have been devised to produce materials with reduced water solubility and greater mechanical strength.^36^

Despite the high efficacy of commonly used aldehyde cross-linkers (formaldehyde and glutaraldehyde), their toxicity raises safety and health concerns, limiting their use, particularly in biomedical and food industries.^32^ Therefore, there is growing interest in the development of more sustainable and safer cross-linking options.^37^ The most common carbodiimide used for coupling biological substances containing carboxyl groups and amines is 1-ethyl-3-(3-dimethylaminopropyl)carbodiimide (EDC) hydrochloride. EDC reacts with a carboxyl group to form an intermediate that reacts with primary amino groups. This carbodiimide is non-toxic and biocompatible.^38^*N*-Hydroxysuccinimide (NHS) stabilizes the amine-reactive intermediate and significantly increases the efficiency of EDC-mediated cross-linking reactions.^38−40^ Indeed, EDC is a zero-length cross-linker that introduces cross-links without the incorporation of foreign structures into the network, e.g., by activating carboxylic acid residues to react with free amine residues, resulting in the formation of an amide bond, without releasing any toxic compound.

EDC cross-linking is bound to be a safer approach than the common use of the bifunctional cross-linkers (formaldehyde, glutaraldehyde, and glyceraldehyde), which are built into the biomaterial and might release toxic compounds into the body upon biodegradation of the hydrogel.^41^

In this work, 3D hydrogels were designed and produced by combining HSs extracted from the compost supplied by Verde Vita (s.r.l.) with gelatin to exploit both physical interactions and chemical cross-linking through EDC chemistry. Rheological tests have been performed to investigate the viscoelastic behavior and strength of the obtained 3D architectures. Furthermore, physicochemical properties of gelatin–HS systems have been assessed through X-ray diffraction (XRD) and attenuated total reflectance (ATR) and nuclear magnetic resonance (NMR) spectroscopies to investigate the interactions between HS and gelatin and their role in conformational and chemical features. Functional characterization has been performed in terms of antioxidant and antimicrobial features to shed light on the possible biotechnological applications of the obtained materials.

## Materials and Methods

### Materials

HSs from the compost supplied by Verde Vita (s.r.l.) (SASSARI, Italy) were extracted following the procedure reported elsewhere.^4^ Briefly, 100 g of air-dried compost samples was suspended in 500 mL of 1 M NaOH solution in polypropylene containers and shaken overnight in a rotatory shaker. The mixture was centrifuged, and the supernatant was neutralized with 37 wt % HCl before being washed again to extract only the fraction of the compost soluble at neutral pH. Then, the solution was dialyzed until Cl-free against distilled water and freeze-dried. Gelatin from porcine skin (300 Bloom grade, type A), EDC, and NHS were purchased from Sigma-Aldrich (Milan, Italy).

### Assessment of Carboxylic Group Content

The percentage of carboxyl groups (COOH) in HS was determined using the potentiometric titration method for humic acids.^42^ A specific amount of 0.1 M HCl was added to a solution containing HS at 8 mg/mL in order to decrease the pH from 7 to 3, favoring the deprotonation of the phenols but not of COOH groups. Then, 0.1 M NaOH was added to the solution until pH 7 [the equivalence point for the acid (COOH of HS)–base (NaOH added) titration] was reached. The percentage of COOH present in the HS under investigation was calculated by evaluating the NaOH moles added. Therefore, EDC moles used were equal to those of COOH. In particular, to obtain cross-linked gelatin solutions, EDC and NHS were added at final concentrations of 2.76 and 1.01 mg/mL (*n*_NHS_ = 0.5 *n*_EDC_), respectively,^41^ to activate carboxyl groups and finally favoring amidic bonds with amino groups.

### Preparation of Neat Gelatin and Cross-Linked Gelatin Solutions

Gelatin powder was dissolved in bidistilled water at 60 mg/mL using a magnetic stirrer at 360 rpm and 60 °C for 30 min to guarantee complete dissolution. Gelatin concentration was chosen in the semi-dilute regime.^43^

### Preparation of Gel HS and Gel HS Cross-Linked Gels

HS solution at 20 mg/mL was prepared by gradually adding and dissolving HS powder in bidistilled water. In particular, the solution was continuously stirred, after each HS addition, for 15 min and sonicated for 10 min. Gel HS solutions were prepared according to the following procedure. Gelatin solutions at different concentrations were prepared, and an appropriate volume was mixed with HS solution at 20 mg/mL to achieve final HS concentrations equal to 3, 8, and 16 mg/mL and a gelatin concentration of 60 mg/mL, as reported in Table 1. The obtained solutions were stirred at 360 rpm and 60 °C overnight. The samples were then stored in glass bottles at 4 °C to get the final gel.

**Table 1:** Composition of the Gel HS Samples

sample	HS composition (mg/mL)	% HS/gelatin (wt/wt)	EDC composition (mg/mL)	NHS composition (mg/mL)
Gel HS 3	3	5.00		
Gel HS 3-EDC	3	5.00	2.76	1.01
Gel HS 8	8	13.33		
Gel HS 8-EDC	8	13.33	2.76	1.01
Gel HS 16	16	26.67		
Gel HS 16-EDC	16	26.67	2.76	1.01
Gel 16 HS 16-EDC	16	100.00	2.76	1.01

Gel HS solutions for chemical cross-linking were prepared in the same way. Then, an appropriate amount of EDC and NHS was added to achieve final concentrations of 2.76 and 1.01 mg/mL, respectively. The final solutions were taken under stirring for few minutes and then stored in glass bottles at 4 °C for 48 h. Then, the obtained gels were washed three times to remove the water-soluble urea derivatives, derived from the presence of EDC.

To investigate a possible cross-linking within the HS supramolecular structure, an additional sample, called HS EDC, was produced. Briefly, HS solution at 8 mg/mL (HS 8) was prepared as previously described. Then, the mixture EDC–NHS at final concentrations of 2.76 and 1.01 mg/mL, respectively, was added. The sample was stored in glass bottles at 4 °C for 48 h and finally washed three times to remove the water-soluble urea derivatives derived from the presence of EDC.

### Dynamic Rheological Measurements

Rheological measurements were carried out in a rotational stress-controlled rheometer (MCR 702, Anton Paar, Linz, Austria) equipped with a Peltier unit for an accurate temperature control (±0.1 °C). In all dynamic experiments, the temperature was kept fixed at 30 °C.

Dynamic frequency sweep tests were performed within the linear viscoelastic range (γ = 5%) by using parallel plate geometry (8 mm diameter, 1 mm gap). To prevent sample drying, a solvent trap was also employed. Samples, already preformed into 8 mm disks, were loaded into the rheometer in the gel-like state.

Dynamic time sweep tests (DTSTs) were carried out in a Couette geometry formed by an outer static cup of 40 mL volume and an inner double-helix spindle (ST24-2HR-37/120, Anton Paar) that is able to mix the solution and measure the rheological properties. To follow in situ gelation, solutions (composed by gelatin and HS) were loaded into the cup in the liquid state and, then, just before the beginning of the test, the cross-linker powder (EDC/NHS) was added.

### X-ray Diffraction

XRD patterns were tracked with a Malvern PANalytical diffractometer (Malvern, U.K.) with a nickel filter and Cu Kα radiation to investigate the crystalline phases of the gelatin protein and its structural changes in the solution due to the addition of HA and/or EDC. The relative intensity was recorded in the range of 2θ from 5 to 80°.

### ATR-FTIR Spectroscopy

ATR-FTIR analysis on both physical and chemical freeze-dried gels was carried out using a Nicolet 5700 FTIR spectrometer (Thermo Fisher, Waltham, MA) by means of a single reflection ATR instrument in the 4000–400 cm^–1^ range with a resolution of 8 cm^–1^ and 32 scans using a ZnSe crystal.

### NMR Spectroscopy

The cross-polarization magic angle spin (CPMAS) technique was applied to perform solid-state NMR spectroscopy of various products. The molecular composition of lyophilized and powdered samples was analyzed on a 300 MHz Bruker AVANCE instrument equipped with a CPMAS wide bore probe using the following acquisition parameters: 10,000 Hz of rotor spin rate, 2 s of recycle time, 1 ms of contact time, 30 ms of acquisition time, and 4000 scans. Fourier transform was performed with a 4 k data point and an exponential apodization of 150 Hz of line broadening.

To enlighten possible modification of structural properties in the synthesized blends, variable spin lock (VSL) experiments have been carried out on different samples with similar instrumental parameters used in an monodimensional experiment except for the stepwise increase of either Spin Lock instrumental times from 0.01 to 10 ms for a total of 16 spectra and for total scans set at 2000 corresponding to 17 h of data collection for each sample. The detected experimental variables were included in eq 1 to estimate the relaxation behaviors of main complex mixtures^44^
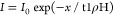
1where *I* is the experimental signal intensity, *I*_0_ is the theoretical max signal intensity, α stands for (1 – tCH/t1ρH), *x* represents the applied instrumental spin lock delays, and t1ρH refers to the molecular proton-lattice relaxation time (in the rotating frame).

### Scanning Electron Microscopy

Scanning electron microscopy (SEM) imaging was carried out on HS, HS-EDC, Gel, Gel-EDC, Gel-HS, and Gel-HS-EDC samples using the following tool: FEI Inspect S; source, 6–12.5 kV; and filament, tungsten equipped with an Everhart–Thornley detector. All samples were dried under vacuum and then sputter-coated with gold before SEM analysis.

### Antimicrobial Tests

Antibacterial activity assays were carried out by two different methods: (1) diffusion disk assay (National Committee for Clinical Laboratory Standards (NCCLS) standard method) and (2) broth microdilution (MIC).^45^ Evaluation of antimicrobial screening was carried out against two Gram-positive (*Listeria monocytogenes667* and *Enterococcus faecalis* ATCC 29212) and two Gram-negative (*Salmonella typhiATCC 19430* and *Escherichia coli* ATCC 25922) bacterial strains. For the first method, the microbial culture was placed in nutrient agar and incubated at 37 °C for 24 h. The inoculum was standardized by transferring colonies from the nutrient agar to sterile saline solution up to 10^8^ cfu mL^–1^ (0.5 McFarland). After this step, an amount of 200 μL of each bacterial cell culture was placed on a Mueller–Hinton agar plate and, at the same time, some disks of 6.0 mm diameter have been employed to test a section of 1 cm of each gel preparation. An antibiotic substance as ampicillin (AMP) was used as a positive control, while bovine serum albumin (BSA), a substance without antibacterial activity, was used as a negative control. The total diameters were evaluated by the analysis of the size (mm) of the inhibition zones. Each experiment was performed in triplicate. The significant difference between mean values was determined by the one-way analysis of variance (ANOVA), and the means (*n* = 3) were validated by applying Tukey’s test at the 0.05 significance level by using the XLSTAT software.^46^ For the second method, the assay was performed in a Mueller–Hinton Broth medium by using sterile 96-well polypropylene microtiter plates. The bacterial cells were inoculated from an overnight culture at a final concentration of about 5 × 10^5^ cfu/mL per well and incubated with all gel samples overnight at 37 °C. Therefore, the minimal inhibitory concentration (MIC) values were estimated by measuring the spectrophotometric absorbance of microtiter plates at 570 nm. The smallest concentration at which no turbidity was observed was considered the MIC value.

### Antioxidant Tests

HS complex supramolecular structures are characterized by the presence of functional groups like quinones and phenolics that can play important roles in biologically relevant redox reactions by acting as natural antioxidants.^47^ The antioxidant activity of both HS and hydrogels was also evaluated by means of the DPPH (2,2-diphenyl-1-picrylhydrazyl) free radical scavenging method^48^ which was carried out according to the following procedure. Briefly, all tests were made by considering the same amount of hydrogels. As regards the samples without HS, used as benchmarks, the antioxidant analysis was carried out considering the same amount of gelatin. The solution containing hydrogels or HS alone was mixed with 2 mL of 100 μM DPPH methanol solution. The mixture was incubated in the dark for 60 and 120 min, and the absorbance at 517 nm was measured by using a UV-2600i UV–VIS spectrophotometer, 230 V (Shimadzu, Milan, Italy).

The percentage of the DPPH free radical scavenging activity is calculated as follows (2)
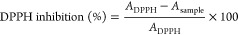
2where *A*_DPPH_ and *A*_sample_ are the absorbance of the methanolic solution of DPPH and the absorbance of the sample, respectively.

### Swelling Analysis

The swelling kinetics of both physical and chemical gels was determined following a well-known method reported in previous studies.^49^ Briefly, the investigated samples were dried under vacuum at 30 °C, weighed, and then rehydrated in distilled water at room temperature. The samples were drained with filter paper to remove water in excess and weighed at different times up to roughly 3000 min. The swelling ratio was defined using eq 3
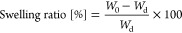
3where *W*_0_ and *W*_d_ are the hydrated and dried weight of the hydrogel, respectively.

## Results and Discussion

### Effect of HS on Viscoelasticity Ramps

**Figure 1:**
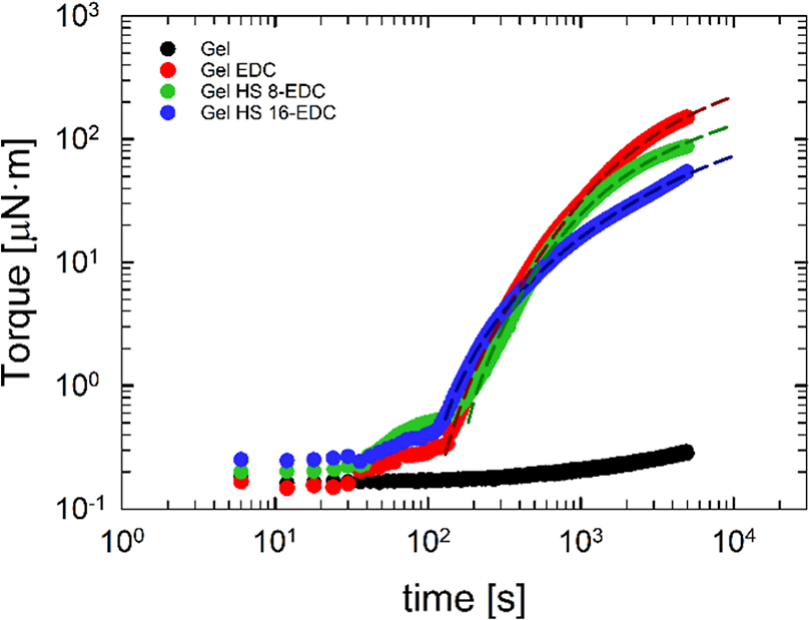
Torque as a function of time during a DTST at 30 °C by fixing ω = 10 rad/s and γ = 5% for gelatin samples (see legend for details). Symbols are experimental data, and lines are fit obtained with eq 4.

Figure 1 shows the torque in the dynamic regime as a function of time for gelatin solutions at different amounts of HS at 30 °C. Figure 1 shows that for the gel sample in the absence of cross-linkers, the torque is almost constant, highlighting that the temperature of 30 °C inhibits the physical gelation, as also proven elsewhere.^43,50^

According to Djabourov et al. (1988),^51^ the physical gelation of gelatin aqueous solutions is the consequence of two processes, as well described by eq 4. The first process is the initial exponential increase of the triple helices with time and the second one is related to the logarithmic growth of the triple helices.^43^ Consequently, eq 4 describes the isothermal formation and growth of the triple helices over time
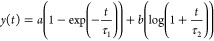
4where *y*(*t*) is the amount of triple helices and *a*, *b*, τ_1_, and τ_2_ are fitting parameters. In particular, τ_1_ and τ_2_ are two characteristic times that govern the formation (and, as such, the initial stages of the gelation process) and growth of triple helices (and, as such, the continuous growth of the gel network), respectively. According to Joly-Duhamel,^52^ the elastic modulus, *G*′, is strictly related to the helices’ amount. Since *G*′ is proportional to the oscillation torque,^53^ we decided to fit the experimental data shown in Figure 1 with eq 4. As a result, the dotted lines in Figure 1 are fits performed when chemical gelation occurs, i.e., after a mixing time of roughly 100 s.

Table 2 reports the values of the regression parameters obtained for each sample.

**Table 2:** Values of the Regression Parameters of eq 4 Used to the Fit Experimental Data Shown in Figure 1

samples	a [μN·m]	τ_1_ [s]	b [μN·m]	τ_2_ [s]
Gel EDC	–65.9∓1.7	456.8∓2.1	255.2∓3.2	805.9∓34.7
Gel HS 8-EDC	–37.1∓2.0	308.6∓2.0	126.3∓2.4	500.0∓42.0
Gel HS 16-EDC	–3.2∓0.1	79.0∓2.1	86.2∓0.7	1503.6∓22.9

It is possible to qualitatively assess the final gel’s strength based on the algebraic sum of the *a*, *b* regression parameters. It can be noticed that when the concentration of HS increases, the gel’s strength slightly decreases.

The τ_1_ values in Table 2 indicate that, upon increasing HS content, the first characteristic time decreases, highlighting that the early stage of the chemical gelation takes place on a shorter time scale when HS is in solution. In other words, higher content of HS promotes the coil to helix formation.

Comparing the values of the second characteristic time, τ_2_, a non-monotonic trend is observed as a function of HS content. Low concentrations of HS (e.g., sample Gel HS 8-EDC) promote the growth of a helical network structure, whereas high amounts (e.g., sample Gel HS 16-EDC) appear to retard the growth of helices. It is possible that, as published elsewhere on a similar system,^49^ at higher concentrations, HS established preferential bonds with water molecules, hindering the triple-helix domains, thus requiring a slower kinetics for their growth.^49^

**Figure 2:**
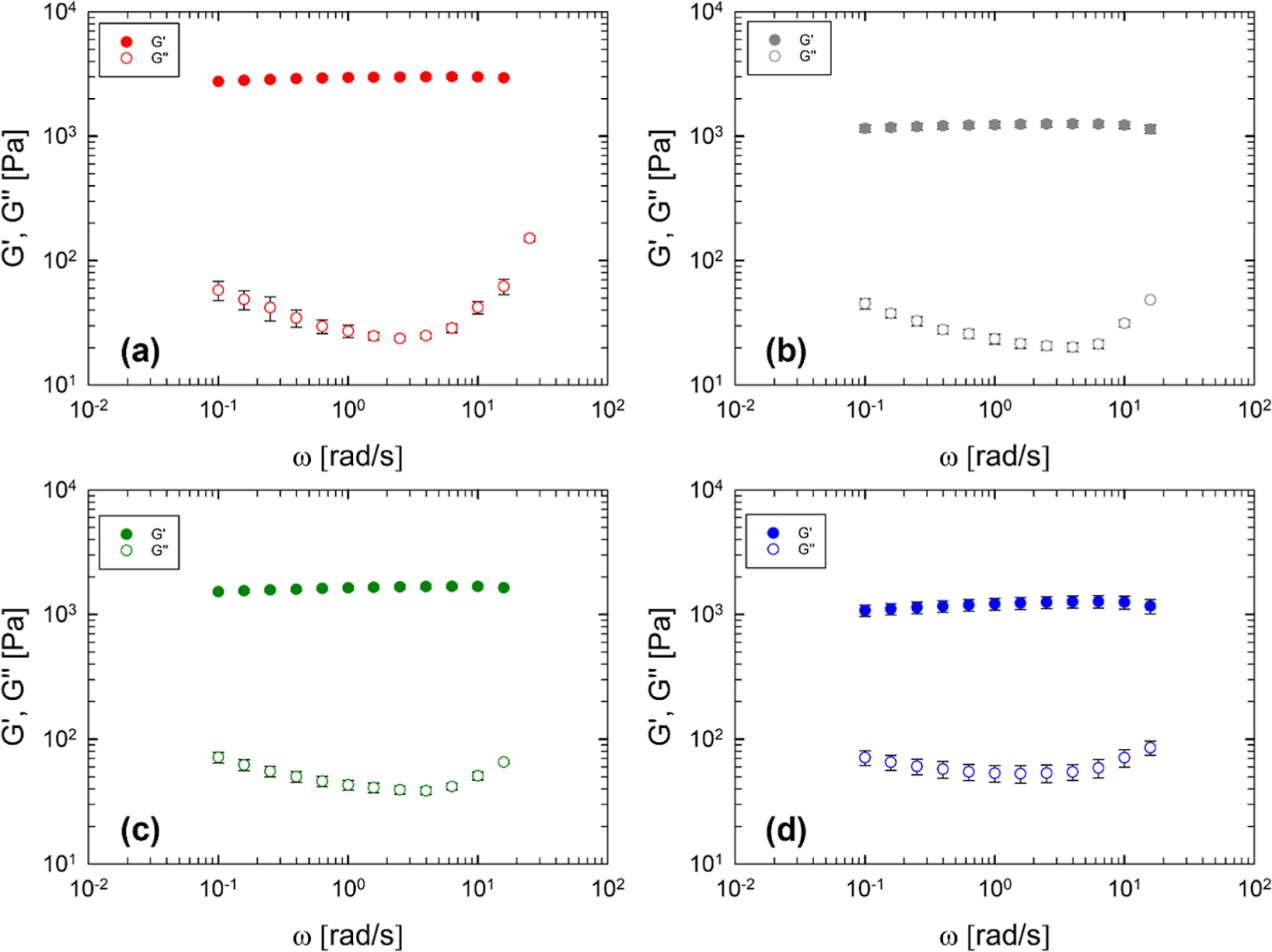
Viscous and elastic moduli as a function of frequency at 30 °C for (a) Gel, (b) Gel HS 3-EDC, (c) Gel HS 8-EDC, and (d) Gel HS 16-EDC. Error bars are shown along with data at different frequencies, as a result of the standard error of multiple measurements (often smaller than data symbols).

Figure 2 displays the dynamic frequency sweep response at 30 °C for gelatin samples with chemical cross-linkers at various HS concentrations. Figure 2 shows the linear viscoelastic behaviors for each sample. Irrespective of the amount of the HS, the rheological response is typical of a gel-like network characterized by *G*′ higher than *G*″ and frequency independent.

**Figure 3:**
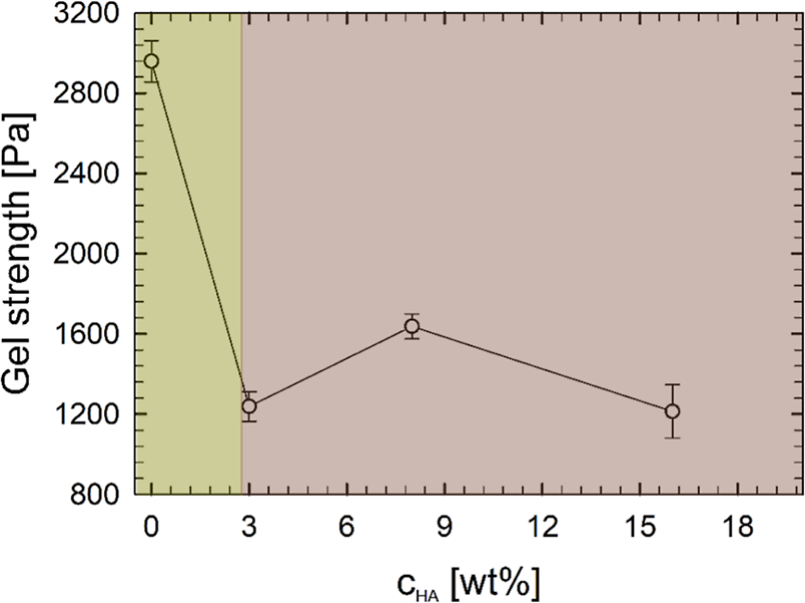
Gel strength (measured as the *G*′ value evaluated at 30 °C at a fixed frequency of 1 rad/s) as a function of relative percentage of HS in solution. Error bars are shown along with the data, as a result of the standard error of multiple measurements.

From a quantitative point of view, Figure 3 shows how the strength of the chemical gel changes according to HS concentration. It is noteworthy that the sample in the absence of HS shows gel strength 3 times higher than that of chemical gels with HSs. Chemical gels with HS show a non-monotonic response for the gel strength with the HS content with a maximum found at a relative concentration of 8% wt. Similar results have been previously achieved on the gelatin/HS system in the absence of chemical cross-linkers.^49^

### Physicochemical Investigation

#### Assessment of Carboxylic Group Content

The assessment of carboxylic group content was carried out on a HS solution at 8 mg/ml according to the procedure described in the Assessment of Carboxylic Group Content section. Indeed, this concentration represents a limit value above which HS biomolecules preferentially interact with water, inhibiting the growth of triple helices and increasing the random coil conformation, as evidenced in a previous work.^49^

The percentage of COOH was calculated by evaluating the NaOH moles added and was equal to about 10% wt with respect to the weight of the HS used in the experiment.

### XRD Analysis

**Figure 4:**
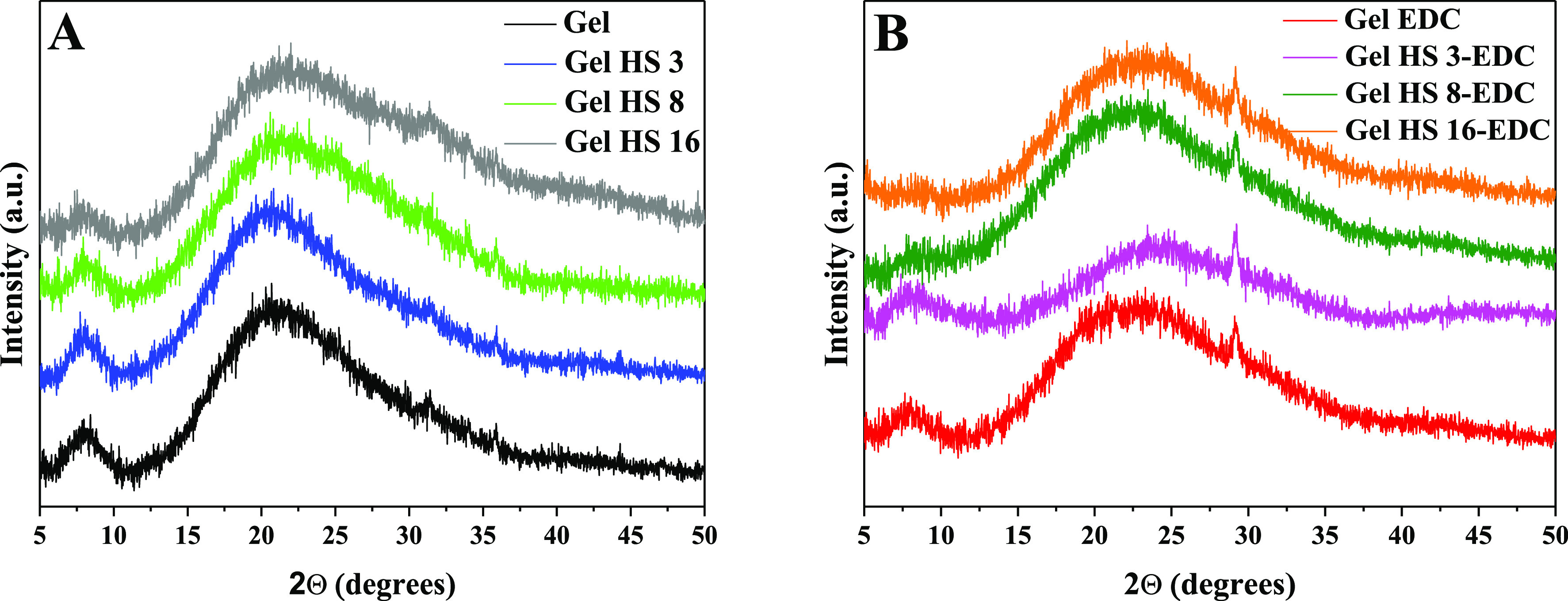
XRD analysis on physical gels (panel A) and EDC-cross-linked gels (panel B).

XRD analysis was carried out to assess the structural changes provoked by HS or EDC addition to gelatin. Figure 4 shows the XRD profiles of physical (Figure 4A) and EDC-cross-linked gels (Figure 4B). The XRD patterns of neat gelatin exhibit a peak 2θ of 8° and a broad halo at 20°, which are usually assigned to an ordered triple-helix and α-helix structure, respectively, thus confirming the partially crystalline nature of the polymer. Indeed, no relevant changes can be appreciated in the XRD patterns of EDC-treated gelatin, suggesting that chemical cross-linking in neat gelatin did not significantly alter its structure, as already reported by previously published studies.^54,55^

However, the peak at 2θ of 8° disappears in the XRD pattern of physical Gel HS samples at higher HS content than 8 mg/ml (Gel HS 8) in accordance with available studies.^49^ This evidence suggests that HSs affect the protein secondary structure, preventing gelatin chains from organizing into triple-helix domains and causing their assembly according to a more disordered organization. As for similar systems reported in the literature, this effect could be ascribed to the high hydrophilic nature of HS moieties, which could preferentially attract the available water molecules and subtract them from H-bond bridging interactions required to form a triple-helix structure.^49^ In addition, more marked changes can be appreciated in the XRD patterns of Gel HS EDC gels. Notably, the peak at 2θ of 8° underwent an abrupt reduction even in the sample containing the lowest HS amount (Gel HS 3-EDC) and completely disappeared at larger HS amounts, thus suggesting that the concurrent addition of HS and EDC strongly influences the protein conformation. From the analysis of XRD results, it can be argued that chemical cross-linking of Gel HS via EDC chemistry destroys the crystal structure, providing the sites to form covalent bonds. Indeed, chemical conjugation is bound to involve amino and carboxylic groups, which react to produce amide linkages. Thus, a decreased number of these moieties could be available for the formation of hydrogen bonds, which consequently get reduced in number, resulting into a less ordered structure.

### ATR Spectroscopy

**Figure 5:**
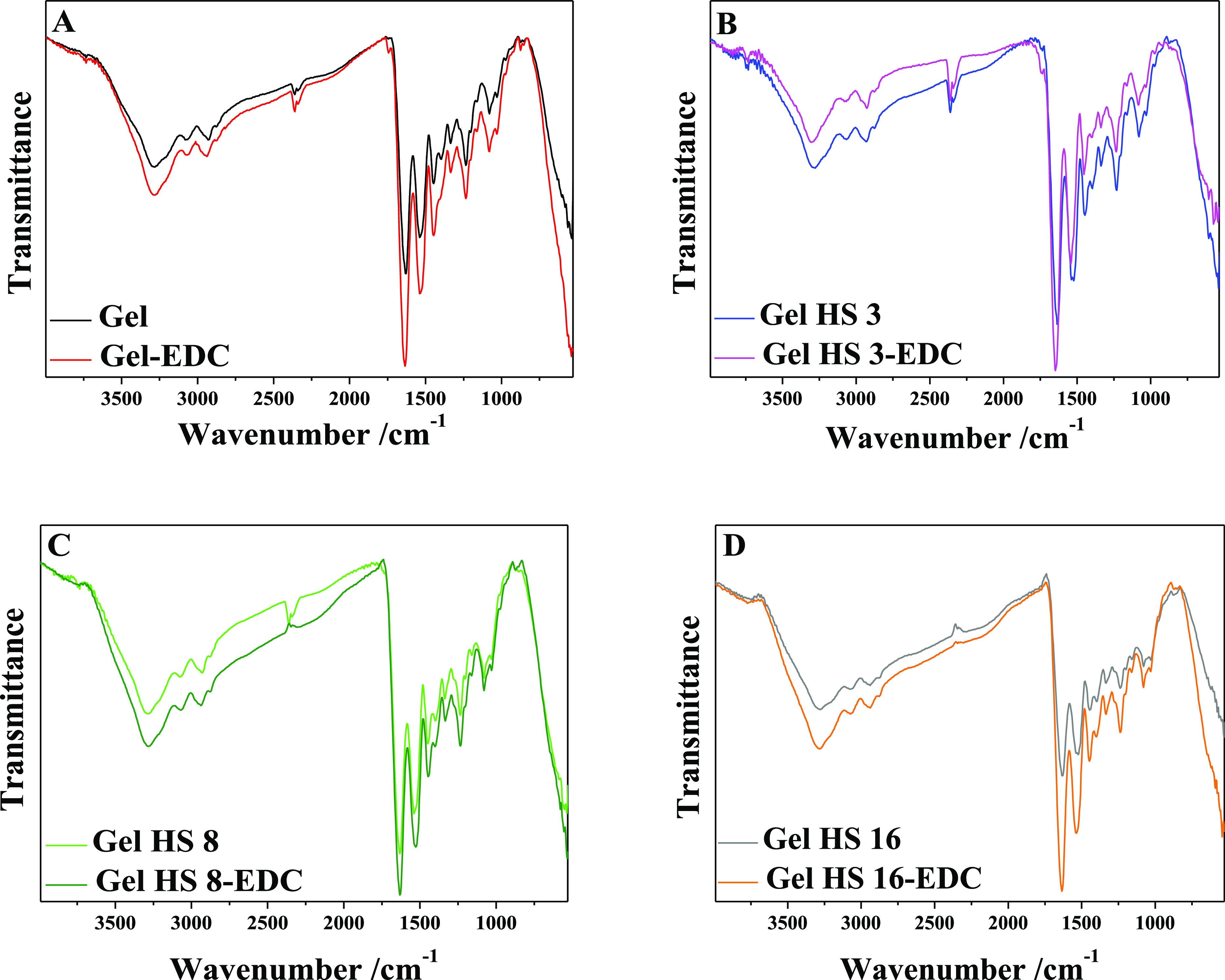
FTIR analysis on physical gels and EDC-cross-linked gels.

The compositional and structural features of the prepared samples were investigated through ATR-FTIR spectroscopy (Figure 5).

The FTIR spectra of the gels exhibited gelatin typical bands, which are listed in Table 3 and include amide A, B, I, II, and III modes among others.

**Table 3:** Infrared Spectral Characteristics of Gelatin

region	wavenumber (cm^–1^)	functional groups
amide A	3430	υ_ΝΗ_, υ_ΟΗ_
amide B	3060	υ_ΝΗ_
amide B	2930	asymmetric and symmetric *υ*_CH2_
amide I	1650	υ_C=O_, υ_ΝΗ_
amide II	1540	δ_ΝΗ_, υ_C–N_, υ_C–C_
amide II	1450	υ_CH_2__
amide II	1410	υ_COO–_
amide II	1330	δ_CH_2__ wagging
amide III	1235	δ_C–N_, δ_ΝΗ_
amide III	1080	υ_C–O_

In particular, the band in the range 3200–3500 cm^–1^ appears as composed of a main absorption peak at about 3300 cm^–1^ and a shoulder at 3280 cm^–1^ as typical NH stretching in primary amines.^56^ No significant changes could be appreciated in the spectra of samples containing HS. This feature might be due to the prevalent gelatin amount in the samples, which hides HS characteristic bands (Figure S1 and Table S1). On the other hand, the FTIR spectra of HS-containing samples show a slight increase in the absorption bands in the region between 2900–3500 and 1500–1900 cm^–1^, which could be better appreciated at higher HS contents (Gel HS 8 and Gel HS 16 samples) and should be straightly related to the presence of HSs, whose main featuring bands fall in the same wavenumber range.^49^

The spectra of gels treated with EDC are similar to those of neat gelatin (Figure 5), thus suggesting that cross-linking with EDC/NHS did not provoke any marked change in the molecular structure, in accordance with previous studies.^55^

At a closer look, the spectra of gelatin films modified with EDC/NHS evidenced a slight increase in the intensity of the amide A, amide I, amide II, and amide III band regions.

The growth of amide A band (3200–3500 cm^–1^, Figure 5) might indicate that EDC cross-linking increases the number of bound NH moieties because of the formation of iso-peptide bonds between the amine groups of gelatin and the activated carboxylic acid groups of either HS or the glutamic or aspartic acid residue of gelatin.^38,57,58^ These findings are in accordance with the increase in the relative intensity of the main absorption band at 3300 cm^–1^ with respect to the shoulder at 3280 cm^–1^, which might reflect the decrease in primary −NH_2_ groups and, therefore, and their conversion into −NH groups because of amide bond formation. Furthermore, the growth of amide I band intensity in EDC-modified gels could indicate an increase in C=O and N–H bond strength, resulting from new covalent bonds in the polymer, thus further supporting the occurrence of chemical cross-linking in EDC-treated samples.^38,57,58^ Moreover, further evidence to the formation of amide groups is provided by the growth of the peak at 1680 cm^–1^.^59^ Finally, a blue shift is observed in amide A, amide I, and amide II bands of chemical gels, suggesting that fewer hydrogen bonds can be established in the samples, as some NH groups could be involved in amide bond formation. These features are more evident in Gel HS samples at high HS content (Gel HS 16-EDC), suggesting that the HS might contribute to chemical cross-linking through amide bond formation.^55^

### ^13^C CPMAS NMR Spectroscopy

**Figure 6:**
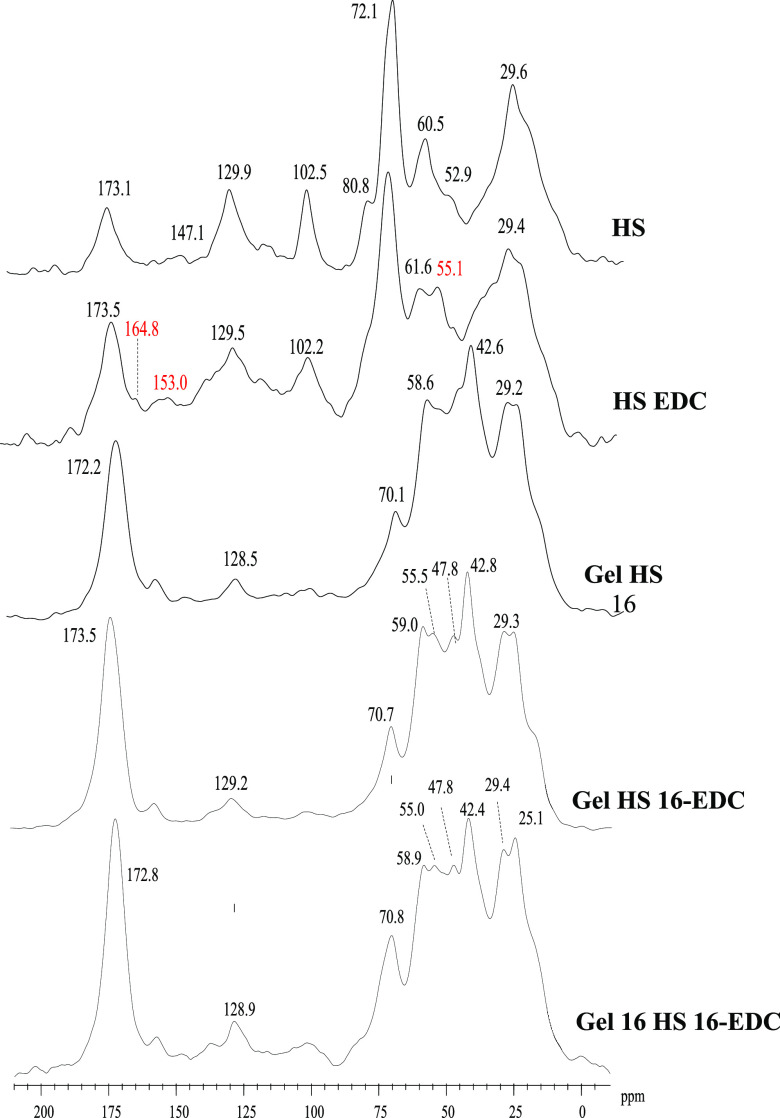
^13^C NMR solid-state spectra of various composite components and gelatin-cross-linked composite blends.

The distribution of the main functional groups of HS, HS EDC, Gel HS 16, and complete blend Gel HS EDC, as detected by the ^13^C CPMAS analysis, is shown in Figure 6. To offset the unavoidable technical low resolution of solid-state NMR spectra, the assignment of resonance bands is based on the split of the overall ^13^C range in six chemical shift intervals which encompass to the most common classes of natural and synthetic organic materials:^60^ 0–45 ppm (alkyl-C); 45–60 ppm (methoxyl-C/C–N groups), 60–100 ppm (O-alkyl-C), 110–140 (Aryl-C), 145–160 (O-Aryl-C), and 160–190 (C=O groups).

The NMR spectra of the HS was characterized by the prevalence of apolar and polar aliphatic components (Figure 6). The band centered at 29.6 ppm includes the CH_2_ building blocks of the alkyl chain of various lipid compounds, while the different resonances in the 60–110 ppm interval are assigned to the C nuclei of pyranoside and furanoside rings of carbohydrates and polysaccharides. The less intense shoulder at 52.9 ppm may be related to the overlapping resonances of methoxyl substituents combined to the C–N bond of peptidic moieties. The broad band in 140–160 ppm derives from the C–C and C–H bonds of aromatic molecules, followed by the O-substituted-C of phenolic derivatives. The final peak at 173 ppm gathers the carbonyl functions of acids, peptides, hemicellulose, etc.

Although the main signals found in the HS EDC sample were mainly determined by the composition of HSs, the inclusion of EDC exhibited slight but evident modification in the C distribution (Figure 6). The small but noticeable peak at 164 in the carbonyl region may be assigned to the formation of amidic bond(s) between the carboxylic groups of HS and the C1 of the EDC sample that in turn give rise to the resonance at 153 ppm. Moreover, the rise in the shoulder at 55 ppm must be associated with the C2 of EDC structures linked to HS molecules. The additional carbons of EDC molecules were incorporated in the alkyl-C fractions, as revealed by the broadening of the band at 29 ppm that highlighted the smoothed shoulders at 33.3 and 38.5 ppm (Figure 6).

The sample made up by a combination of gelatin with HS, Gel HS 16, was dominated by the C nuclei of peptide chains (Figure 6). The resonances in the 45–60 ppm region are assigned to the αC–N of amino acids whose side chains produced the intense peaks recorded in the chemical shift interval of alkyl-C (0–45 ppm) identified by the signals at 25 and 29 ppm.^61,62^ The peak at 70 ppm may be assigned to the C4 of Hyp amino acid^61^ combined with the possible contribution of O-alkyl-C from HS, while the signal around 128 ppm combines the aryl-C of aromatic amino acids. Finally, the sharp band at 172/173 ppm underlines the presence of C=O groups of amino acids in either an acidic function or involved in the amidic bonds. The NMR spectra of the complete blends Gel HS 16-EDC and Gel 16 HS 16-EDC, synthesized by the interaction of gelatin with the HS–EDC complex, were still marked by the predominance of the peptidic functional groups (Figure 6). However, the new emerging peaks at 48 and 55 ppm, found in both blend samples, suggest the possible inclusion of additional αC of amidic bonds between the NH_2_ groups of gelatin and the activated C=O functions of the HS–EDC complex (Figure 6). The relative increase in the two peaks with respect to the contiguous signal of original gelatin components at 59 and 42.5 ppm in Gel 16 HS 16-EDC, formed with a lower amount of gelatin, further supports the involvement of new binding interactions between gelatin and HA, which actively concur chemical gel formation.

The structural properties of composite samples were further assessed by the evaluation of cross-polarization dynamics, performed with pseudo-bidimensional solid-state VSL NMR experiments. The sensitivity of the solid-state NMR technique is based on ^1^H–^1^H and ^1^H–^13^C dipolar nuclear interactions and structural mobility, which largely determine the magnetization features and the cross-polarization (CP) behavior.^63^ In complex organic macromolecules, the intensity, resolution, and relaxation trend of various signals are strictly influenced by proton density and steric proximity of high- and low-protonated functional groups that may allow averaging and matching of cross-polarization parameters with a sharing of magnetization properties (spin diffusion). However, the variation of local chemical surrounding may modify the response of CP parameters, such as the time delays required for both the complete ^1^H–^13^C polarization transfer to maximize the signal intensity (cross-polarization time) and the decay of proton magnetization (spin–lattice proton relaxation time-t1ρH). The estimation of these variables may be used to infer the conformational properties related with the spatial homogeneity and conformational constraints of molecular domains.^44,64^

**Figure 7:**
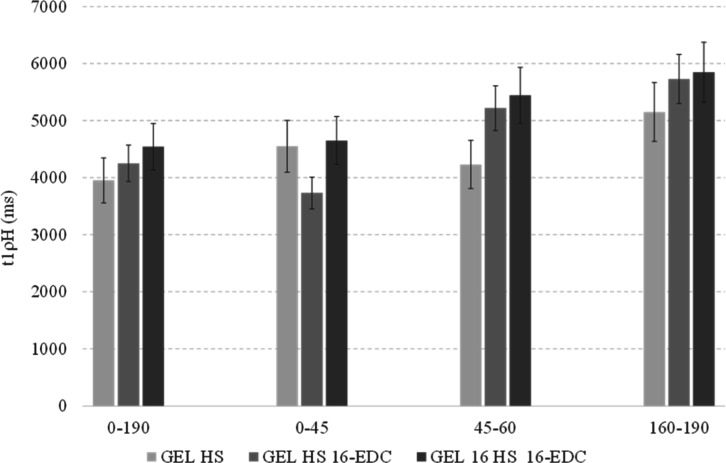
Relaxation times (t1ρH) of Gel HS and Gel HS-cross-linked blends determined by VSL 13C NMR experiments.

Figure 7 indicates the estimated spin–lattice proton relaxation time (t1rH) (Figure S2a) derived from VSL experiments (Figure S2b) performed on Gel HS 16, Gel HS 16-EDC, and Gel 16 HS 16-EDC, as related to the chemical shift on different C functionalities, while the corresponding relaxation curves are included in Figure S2 of the Supporting Information.

The average relaxation times detected for the identified functional groups of gelatin structure are in line with the values reported in previous analysis recorded with variable contact time experiments.^61^ The almost uniform relaxation dynamics (Figures 7 and S2) suggested an effective mixing of spectroscopic properties. The presence of contiguous protonated molecules in the side chains of peptic components promoted a homogeneous spin communication system and a consequent sharing of CP properties. Notwithstanding the even CP behavior, a slight increase in t1ρH was detected in the 45–60 ppm chemical shift range (Figure 7). This finding may be connected to the hypothesized formation of additional amidic bonds in a composite blend between gelatin and humic additive that may have stiffen the local conformational arrangement and reduced the molecular mobility, thus slackening the decaying rate of magnetization intensity.^44^

As evidenced by FT-IR and NMR spectra, the main functional groups of HS include carboxy and catechol moieties, which make them similar to polyphenols. These compounds are bound to bind to proteins through different mechanisms.

Under oxidizing conditions, quinone moieties produced by catechol oxidation can react with the amino groups of proteins via a Schiff base reaction and Michael addition, leading to Schiff bases (C=N) and Michael adducts (C–NH–R), respectively.^65−67^

In view of the chemical similarity between HS and polyphenols, it can be inferred that the chemical cross-linking pathways described above could account for their covalent interaction with gelatin in view of their chemical similarity between HS and gelatin.

In addition, chemical grafting can occur through the amide bond between the carboxyl groups of HSs and the amino groups of gelatin via EDC chemistry.^68^

From NMR and FT-IR spectroscopic evidence, it can be argued that HS and gelatin essentially interact through amide bond formation. Indeed, the survey of the CPMAS NMR spectra of raw HS, HS EDC, and Gel HS EDC did not highlight any relevant additional peaks related to other possible chemical coupling pathways between HS and gelatin. Thus, it can be inferred that chemical binding via EDC chemistry mainly accounts for chemical gelation.

### SEM Analysis

**Figure 8:**
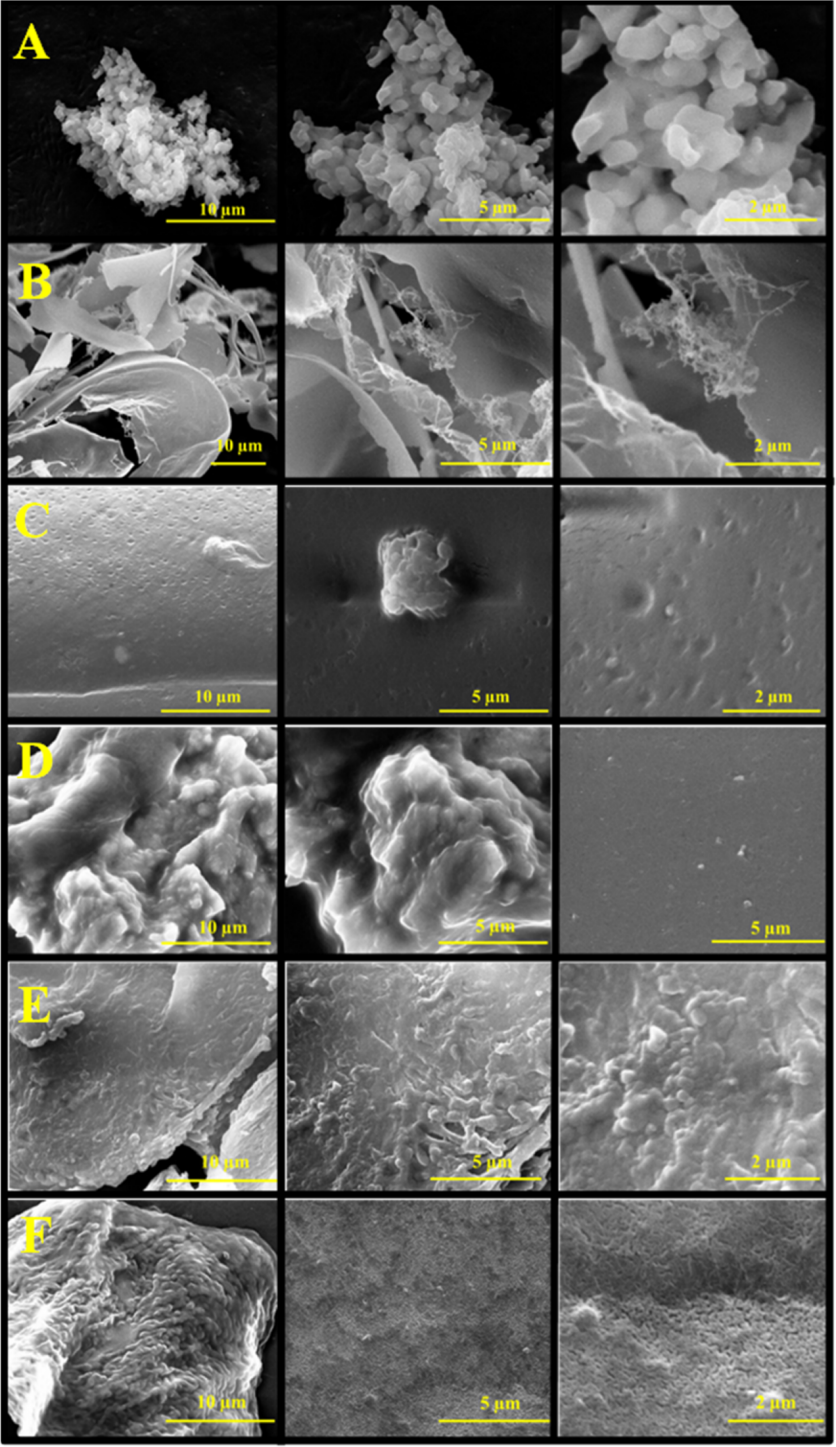
SEM images of (A) HS; (B) HS EDC; (C) Gel EDC; (D) Gel HS 3-EDC; (E) Gel HS 8-EDC; and (F) Gel HS 16-EDC.

The SEM micrography of the neat HS shows the presence of irregular micrometric aggregates (Figure 8A), which looked as smaller primary particles and might have precipitated during sample drying. On the other hand, HS EDC (Figure 8B) underwent a significant morphology change, evidencing a sheet-like appearance in SEM pictures.

Indeed, chemical cross-linking of HS through EDC chemistry, as suggested by NMR spectra, is bound to improve the water sorption ability,^69^ which might result in highly swelled wet samples exhibiting an unfolded structure upon drying.

**Scheme 1:**
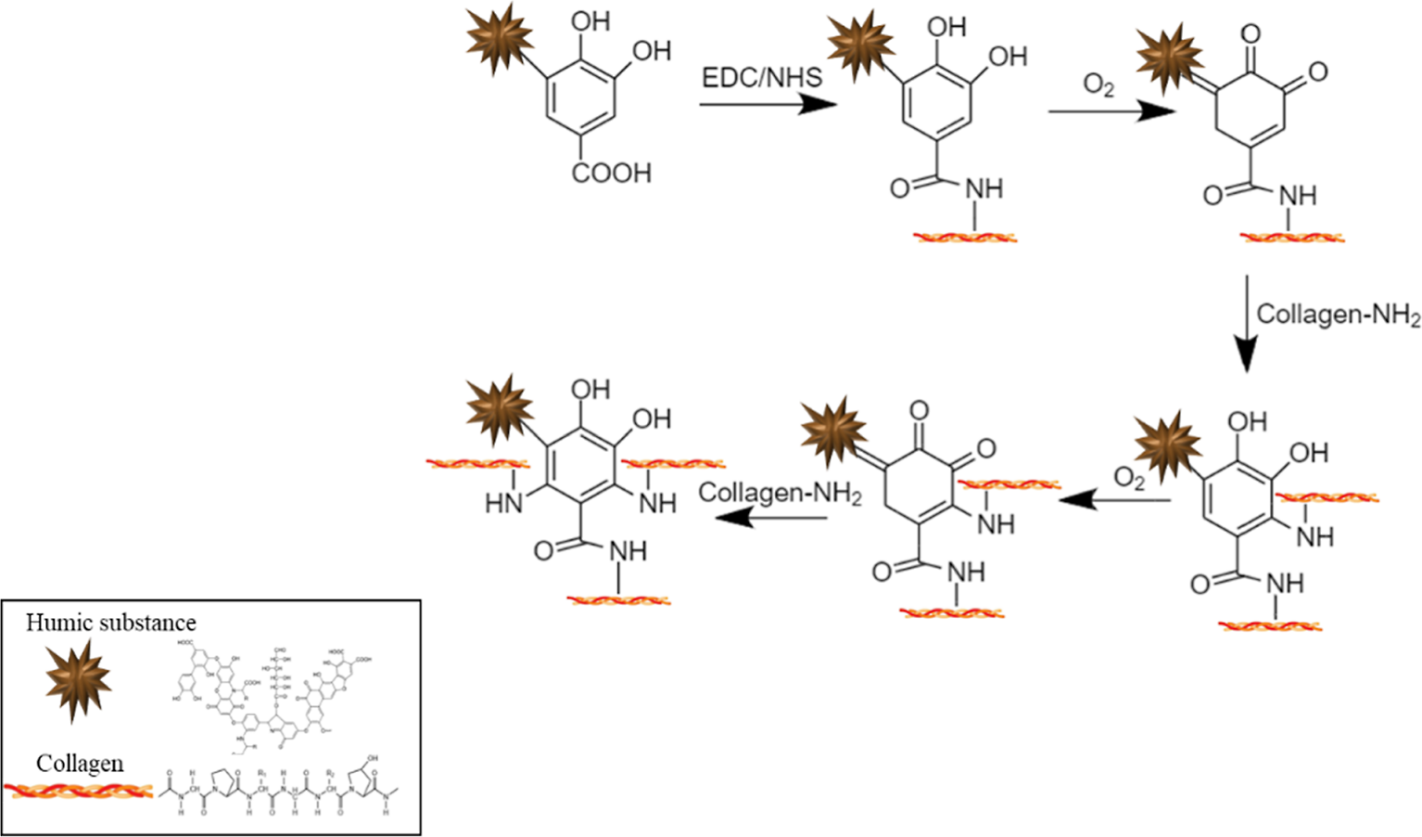
Chemical Cross-Linking between Activated Carboxyl Groups of HS and Gelatin by Means of the EDC/NHS Complex

The SEM pictures of neat gelatin samples evidence a typical smooth surface.^49^ On the other hand, the addition of HS caused the built up of a rough structure because of the formation of submicrometric particle aggregates, the larger amount of the higher HS content, in accordance with previous studies^49^ (Figure 8D–F). Indeed, these clusters could be coacervates, obtained by partial protein coagulation phenomena, provoked by protein–HS interactions. A similar behavior was already observed by the addition of polyphenols, which caused gelatin coacervate formation.^70,71^ At a closer look, the observed aggregates appeared smaller and more abundant in EDC-treated samples than in the physical gels with a similar composition.^49^ This result could be an indirect proof that a tighter interaction can be established between HS and gelatin via EDC chemistry. Polyphenols, including gallic acid, are bound to interact with collagen chains through hydrophobic and H-bond interactions.^72^ In addition, EDC treatments enable chemical cross-linking through amide bond formation as well as quinoid reaction of GA and collagen.^72^ Similar interactions could be even established between HS and gelatin, according to Scheme 1, in view of HS chemical similarity with polyphenol moieties. Tighter chemical cross-linking might promote gelatin coagulation, leading to the formation of a higher number of smaller aggregates than those present in physical gel, accounting for the morphology evidenced in the SEM micrographs of Gel HS-EDC.

### Functional Assessments

#### Antimicrobial Tests

Microbial infections caused by different kinds of pathogens threaten human health. For instance, various medical devices such as implants, contact lenses, and catheters are prone to carry pathogenic microorganisms caused to microbial infection.^73^ Microbial resistance strategies against medicine are promoted day by day. Bacterial organisms can protect themselves from superficial physical and chemical disinfection using different protocols including biofilm formation, change in the physical properties of mature biofilms, alteration in the genotype of the bacteria, production of neutralizing enzymes, and physiological attributes within biofilms.^74^ Antibacterial polymers can prompt the effectiveness and specificity of applied antimicrobial agents while reducing the associated environmental risks because these polymers have non-permeable properties and are chemically stable.^75^

Among the various antimicrobial agents, bioavailable moieties, including biopolymer gels, are good alternatives to antibiotics, and this may be due to their multi-biological function as well as their compatibility.^76^ HSs represent the most extensive and reactive class of the natural compounds which are a part of the organic substances of soils, natural waters, and solid combustible minerals but also we can obtain these materials starting from wastes which can be treated as a source of bioactive compounds. HSs have found wide application also in the fields of medicine or veterinary sector^13,45,77−80^ thanks to their antiviral, antimicrobial, profibrinolytic, anti-inflammatory, and estrogenic activities. In this context, some research shows that fulvic acids, a HS component, have the power to protect against cancer and related cancer-causing viruses.^81,82^ The antibacterial features of different prepared gels were investigated by the diffusion disk assay and broth microdilution (MIC).

**Figure 9:**
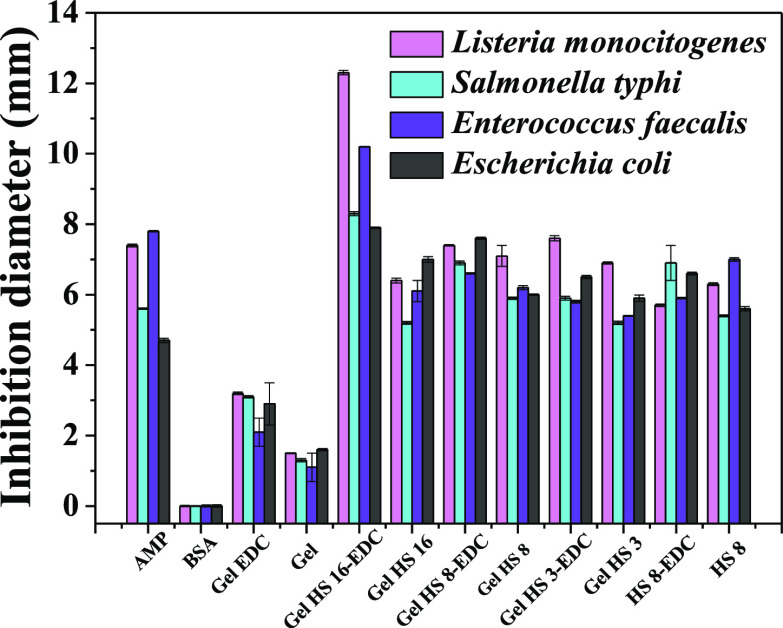
Antibacterial activity of gelatin-based physical and chemical gels combined with HS against Gram-negative and Gram-positive bacterial strains. The error bars indicate the standard error (*n* = 3); the standard deviation was less than 5%.

The antimicrobial power results of both physical and chemical gels are reported in Figure 9 and Table 4.

**Table 4:** Antibacterial Activity of All Samples as MIC against Some Gram-Positive and Gram-Negative Bacterial Strains^a^

MIC (μg·mL^–1^)	Listeria monocytogenes	Salmonella typhi	Enterococcus faecalis	Escherichia coli
AMP	0.01 ± 0.1	0.04 ± 0.02	0.03 ± 0.2	0.05 ± 0.01
BSA	n.i.	n.i.	n.i.	n.i.
Gel EDC	132 ± 0.02	139 ± 0.01	129 ± 0.05	125 ± 0.07
Gel	149 ± 0.03	140 ± 0.5	135 ± 0.6	122 ± 0.1
Gel HS 16-EDC	3.4 ± 0.1	2.8 ± 0.02	4.5 ± 0.7	5.8 ± 0.03
Gel HS 16	4.5 ± 0.4	4.9 ± 0.06	6.7 ± 0.8	8.2 ± 0.06
Gel HS 8-EDC	16.2 ± 0.6	21.4 ± 0.2	23.5 ± 0.06	24.6 ± 0.04
Gel HS 8	18.1 ± 0.07	23.5 ± 0.07	27.4 ± 0.01	28.5 ± 0.05
Gel HS 3-EDC	32.5 ± 0.03	39.4 ± 0.03	37.1 ± 0.5	43.6 ± 0.07
Gel HS 3	37.3 ± 0.02	38.6 ± 0.01	41.8 ± 0.02	51.4 ± 0.01
HS 8-EDC	17.2 ± 0.1	24.5 ± 0.07	24.1 ± 0.08	26.1 ± 0.02
HS 8	18.1 ± 0.4	25.2 ± 0.3	28.2 ± 0.01	29.1 ± 0.01

a The MIC values shown in the table are the highest concentrations obtained after three independent experiments. n.i., no inhibition, overgrowth of bacterial cells on the platelets.

All hybrid gels obtained using HSs showed a strong antimicrobial activity compared to that of bare gelatin. The prepared samples exhibit widespread biocide action toward both Gram(+) and Gram(−) strain. The antimicrobial activity in terms of DDK results is at least comparable to that of AMP used as a control and even to neat HS at 8 mg/ml (HS 8), yet with a lower content in the active component. Similar results have been obtained by the assay with MIC, as shown in Table 4.

Indeed, the combination of HS with gelatin preserves their intrinsic antibacterial activity. This is bound to be exerted through the synergy between bacterial membrane destabilization and ROS production.^5,15^ Notably, carboxylic and phenolic groups in HS could increase the number of hydrogen bonds with cell bacterial membranes.^15^ This higher affinity should induce irreversible modifications in its lipid structure, enhancing the permeability of ROS, which are produced by quinone redox equilibria in HS, with a consequent reduction in pathogenic strain growth and their death.^15^ Apparently, the porous structure and hydrophilic features of the matrix ensure the exposure of active moieties and enable their interaction with the bacterial membrane. At the same time, they assure an aerated wet environment required for ROS production by quinone redox chemistry.

The obtained results showed that the antimicrobial performance improves by increasing the HS amount within the hybrid gels. In addition, gels treated with EDC exhibit a higher inhibition diameter^74^ and lower MIC values compared to those of the corresponding physical gels, suggesting that the hybrid modification with HS using EDC chemistry allows for the reduction in their water absorption and gives them better antibacterial properties.^83^ Indeed, the higher antimicrobial capacity of humic gels was related to the chemical cross-linking between gelatin and HS. Notably, larger water uptake determined by HS cross-linking at large HS content might enable better exposure of active moieties and determine a more effective biocide action.^84^

Several studies prove biocompatibility and bioactivity of HSs.^5^ These moieties have been exploited as biostimulants to promote plant growth. In addition, their intrinsic safety has stimulated testing as bioactive compounds in biomedical applications.^85^

At the same time, gelatin is a natural polymer that is biodegradable, biocompatible, and non-immunogenic and has a low coagulation activity toward platelets. These properties make gelatin a suitable compound for biomedical applications.^41^

Indeed, EDC cross-linking is bound to be a safer approach than the common use of the bifunctional cross-linkers (formaldehyde, glutaraldehyde, and glyceraldehyde), which are built into the biomaterial and might release toxic compounds into the body upon biodegradation of the hydrogel.^41,86^ Thus, it can be argued that chemically cross-linked gelatin-HS hydrogels keep the non-cytotoxic features of their components.

#### Antioxidant Tests

The antioxidant assay was performed using a fixed amount of hydrogel in contact with the DPPH methanol solution for 1 or 2 h. Table 5 displays the antioxidant activity in terms of DPPH inhibition of both physical and chemical gels after 1 and 2 h. It is possible to observe that HS plays an important role in conferring antioxidant power to the obtained gels; indeed, HS complex supramolecular structures are distinguished by the presence of functional groups including quinones and phenols, which can play important roles in biologically relevant redox reactions by acting as natural antioxidants.^47^

**Table 5:** Antioxidant Results of Chemical Gels at *t* = 1 h and *t* = 2 h

sample	DPPH inhibition @1 h (±2%)	DPPH inhibition @2 h (±2%)
Gel	35	40
Gel EDC	50	60
Gel HS 3	67	78
Gel HS 3-EDC	76	80
Gel HS 8	82	90
Gel HS 8-EDC	87	89
Gel HS 16	90	95
Gel HS 16-EDC	90	95

In our case, the sample with the lowest HS content shows an increase in antioxidant activity of about 30% with respect to bare gelatin. Physical and chemical hybrid gels have a strong DPPH inhibition thanks to the presence of HS, even if at lower HS content chemical gels have a slightly higher antioxidant activity at 1 h. After 2 h of contact between the sample and the DPPH solution, the gel with the highest amount of HS (gel HS 16-EDC) has the highest antioxidant activity of approximately 95%.

### Swelling Ratio

**Figure 10:**
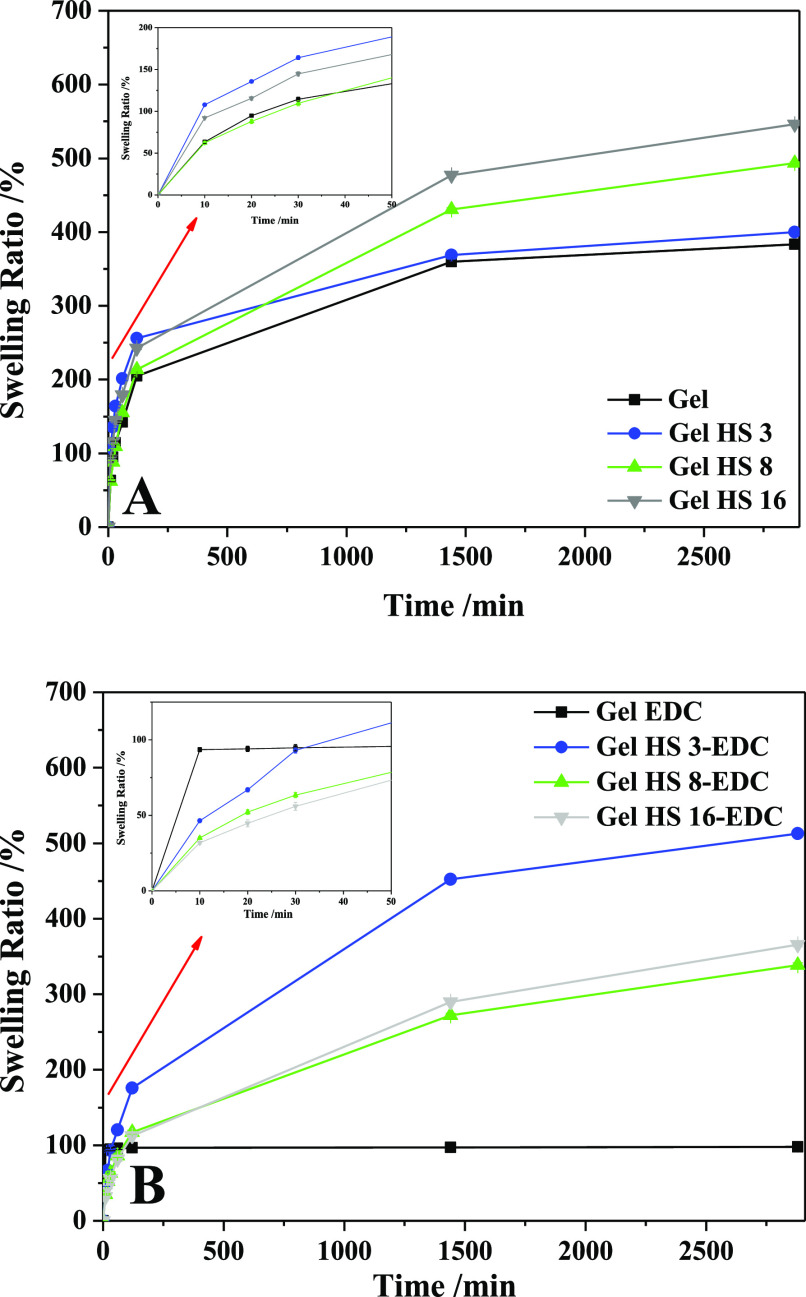
Swelling analysis on physical gels (A) and chemical gels (B).

Figure 10 displays the swelling ratio of neat gelatin (Gel) and hybrid gel (Gel HS) samples. In physical gels, the swelling ratio of Gel and Gel HS 3 samples (Figure 10A) steadily increased, reaching a constant value of about 400% after about 1500 min, whereas Gel HS 8 and Gel HS 16 increased their swelling ratio after 48 h up to a value of about 500%. As a result of HS ability to absorb water, its addition resulted in an increase in swelling ratio. Indeed, HS exhibits a distinct hydrophilic behavior, as shown by the relevant swelling phenomena in water, and demonstrated in our previous work, and this feature could improve the water sorption and swelling behavior of gelatin.^49^ On the other hand, a non-monotonic trend was observed for the swelling ratio of chemical gels (Figure 10B). More specifically, bare gelatin (Gel EDC), despite having the highest swelling kinetics, reached a constant swelling ratio of about 100% after 10 min, demonstrating the effect of complex EDC/NHS cross-linking. The sample with the lowest HS content (gel HS 3-EDC) has the greatest swelling kinetics compared to the other hybrid gels and the highest swelling ratio due to the HS hydrophilic behavior, which in this case predominates over the EDC/NHS complex cross-linking effect. Instead, higher HS content samples (gel HS 8-EDC and gel HS 16-EDC) have a swelling ratio that is lower than that of HS 3-EDC gel but higher than that of chemically cross-linked neat gelatin.

Overall, the swelling ratios of Gel HS samples improved with respect to bare gelatin when the cross-linking agent was introduced. The swelling ratio of chemically cross-linked hydrogels are influenced by the hydrophilic nature which increases with HS content and cross-linking degree. The higher the former, the larger the water absorption. On the other hand, the higher the cross-linking density, the lower the swelling. The decrease of water absorption ability with respect to the physical gel could be related to the presence of amide chemical bridges in neat gelatin treated with EDC (Gel EDC). Indeed, cross-linking and hydrophilicity are strongly related in HSs. These are considered as supramolecular structures obtained by self-assembly of relatively small and heterogeneous molecules, held together by weak dispersive forces, including van der Waals, π–π, and H-bond interactions. Water absorption can determine a collapse of these structures because of the break of H bonds. On the other hand, the presence of covalent bonds can stabilize these supramolecular organizations, which are no longer broken upon swelling. Accordingly, water sorption of HSs improves with covalent cross-linking.^84^ This phenomenon could occur in samples containing large HS amounts (Gel HS 16-EDC) because of the EDC treatment and result in larger water holding ability, which might counterbalance improved cross-linking density in gelatin hydrogel.

It appears that gelatin-based 3D hydrogels cross-linked with EDC are still biodegradable even though the biodegradation kinetics can be affected by the cross-linking degree. Several studies confirm this evidence, but different results have been obtained for the degradation time, which is affected by the procedure employed in degradation tests. For example, cross-linked fish gelatin films can be generally degraded at a slower rate with respect to the non-cross-linked films but eventually degraded at a faster rate after 60 days.^87^ On the other hand, a previous study, evaluating degradation through weight loss measures, showed that porcine gelatin films modified with various cross-linkers degraded faster in soil than the physical film.^88^

In any case, a slower biodegradation rate does not necessarily represent a negative aspect. Indeed, the ability to control the chemical and physical properties of the gel through cross-linking chemistry has allowed us to obtain a gel with superior antimicrobial properties compared to a physical one. Besides, cross-linking could increase the stability of the gel under environmental conditions, improve the mechanical strength and flexibility of the gel, and enhance its ability to release active principles in a controlled manner.

## Conclusions

In this work, hybrid gelatin hydrogels have been prepared at different HS contents, and their physicochemical, rheological, and functional properties have been analyzed.

The physical blending or chemical combination through EDC chemistry between HS and gelatin from porcine skin is an effective and promising strategy to turning HS incoherent and heterogeneous powders into self-standing and mechanically stable 3D materials.

The morphological, physicochemical, and rheological analysis of the obtained materials allowed us to demonstrate the type of interaction established between biowaste and gelatin. In particular, ATR and NMR spectroscopies unveiled the chemical interaction between the two components, proving that the presence of EDC promoted amide bond formation between the amino groups of gelatin and the activated groups C=O of the HS–EDC complex. This affects the gel formation kinetics as well as protein conformation, as proven by rheological tests, evidencing a different rate of formation and growth of helix domains due to HS content. Besides, chemical cross-linking between gelatin and HS, as well as within HS supramolecular structures, allowed higher swelling ratios than neat cross-linked gelatin and enabled better exposure of bioactive moieties, without any dissolution phenomena. This results in a significant improvement of antimicrobial behavior, with respect to neat HS and physical gels.

From the technological point of view, this study proposes a straightforward and sustainable strategy to add value to commercial biowastes, as HSs, by converting them into 3D active gels. This strategy could be easily upscaled and extended to other biowaste compositions. From the scientific point of view, this manuscript provides evidence that chemical cross-linking improves the intrinsic features of HSs and provides an explanation to this not trivial outcome. These features could be highly relevant for the effective management and valorization of HSs, which are one of the most abundant biowastes.

The manuscript was written through contributions of all authors, and all authors have given approval to the final version of the manuscript.

## Supporting Information Available

FTIR spectrum of HS; assignment of FTIR bands of HS; examples of exponential decay curves derived from ^13^C CPMAS VSL experiments; and representative ^13^C CPMAS NMR VSL experiments (PDF)
