# Supplementary material for: Waste to Wealth Approach: Improved Antimicrobial Properties in Bioactive Hydrogels through Humic Substance–Gelatin Chemical Conjugation
Source: Biomacromolecules. 2023 May 11;24(6):2691–705. doi: 10.1021/acs.biomac.3c00143 (PMC10265667; doi:10.1021/acs.biomac.3c00143)
Supplement: Supplementary file 1 — bm3c00143_si_001.pdf [file bm3c00143_si_001.pdf]

Supporting Information for Publication

A waste to wealth approach: improved antimicrobial properties in bioactive hydrogels through humic substances-gelatin chemical conjugation

Virginia Venezia [1,2], Mariavittoria Verrillo [3], Pietro Renato Avallone [1], Brigida Silvestri [4], Silvana Cangemi [3], Rossana Pasquino [1], Nino Grizzuti [1], Riccardo Spaccini [3] and Giuseppina Luciani [1]**

[1] DICMaPI, Department of Chemical, Materials and Industrial Production Engineering, University of Naples Federico II, Naples, Italy

[2] DiSt, Department of Structures for Engineering and Architecture, University of Naples Federico II, Naples, Italy

[3] Department of Agricultural Science, University of Naples Federico II, Portici, Italy

[4] Department of Civil, Architectural and Environmental Engineering, University of Naples Federico II, Naples, Italy

*Corresponding authors: luciani@unina.it (G.L.), riccardo.spaccini@unina.it (R.S.)

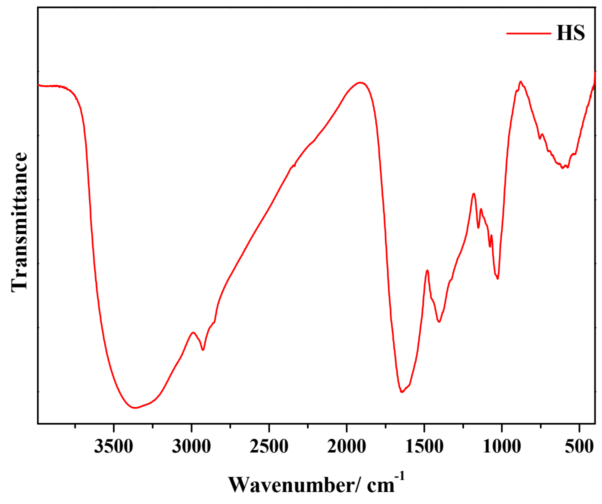

Figure S1. FTIR spectrum of HS.

Table S1. Assignment of FTIR bands of HS.

**Bands (cm^{-1})**	**Assignments**
3700-3000	Stretching vibration of OH
2800-3000	Symmetric and asymmetric vibration of $-CH_2$
1580-1760	C=C (aromatic and olefins), C=O (ketones and quinone)
1400	-OH (phenols), COO, and $-CH_3$ bending
1280-1020	C-O and C-O-R structures
540-460	Minerals

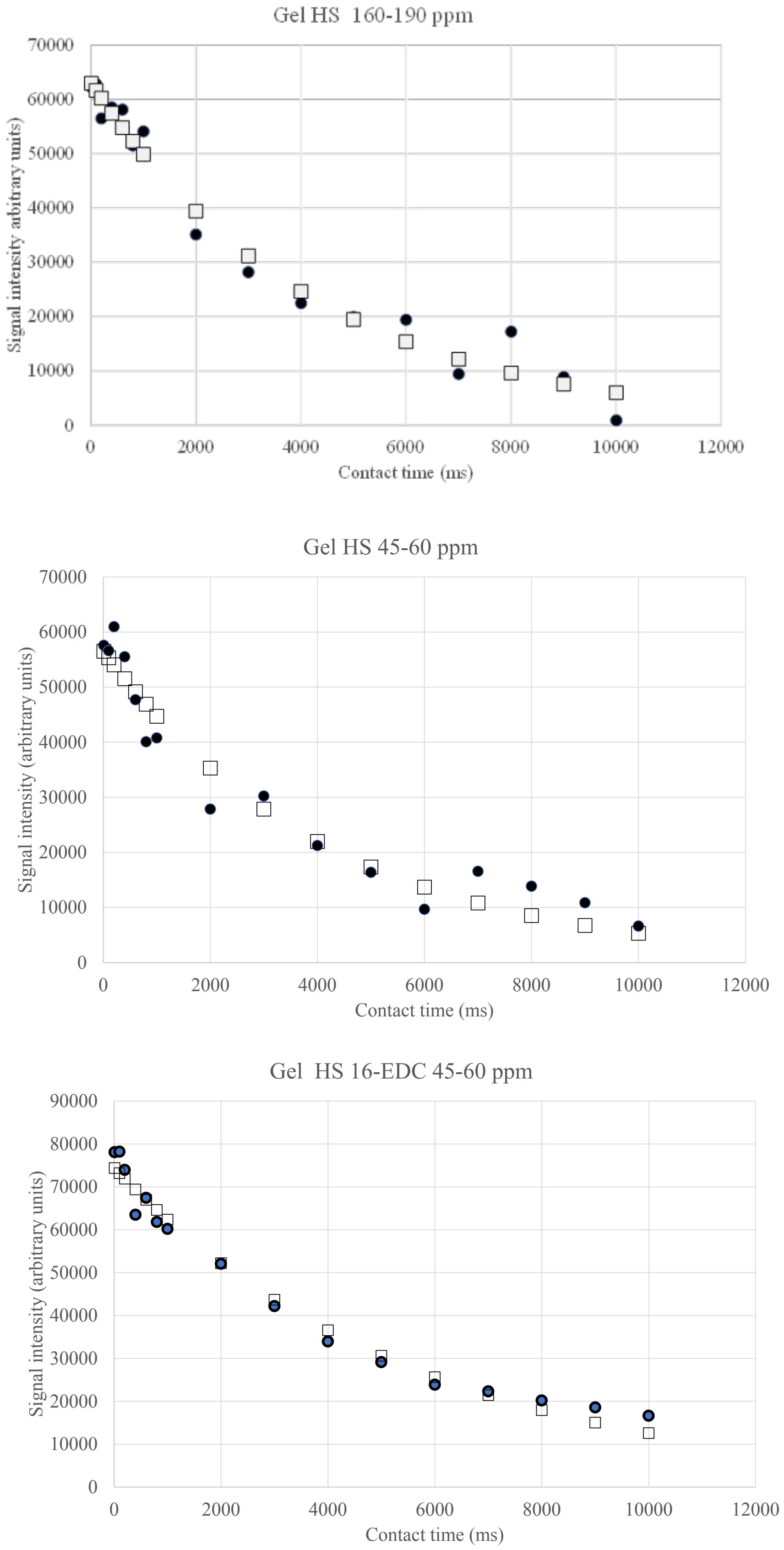

Figure S2a. Examples of exponential decay curves derived from ^{13}C CPMAS VSL experiments.
• experimental data □ fitted curve

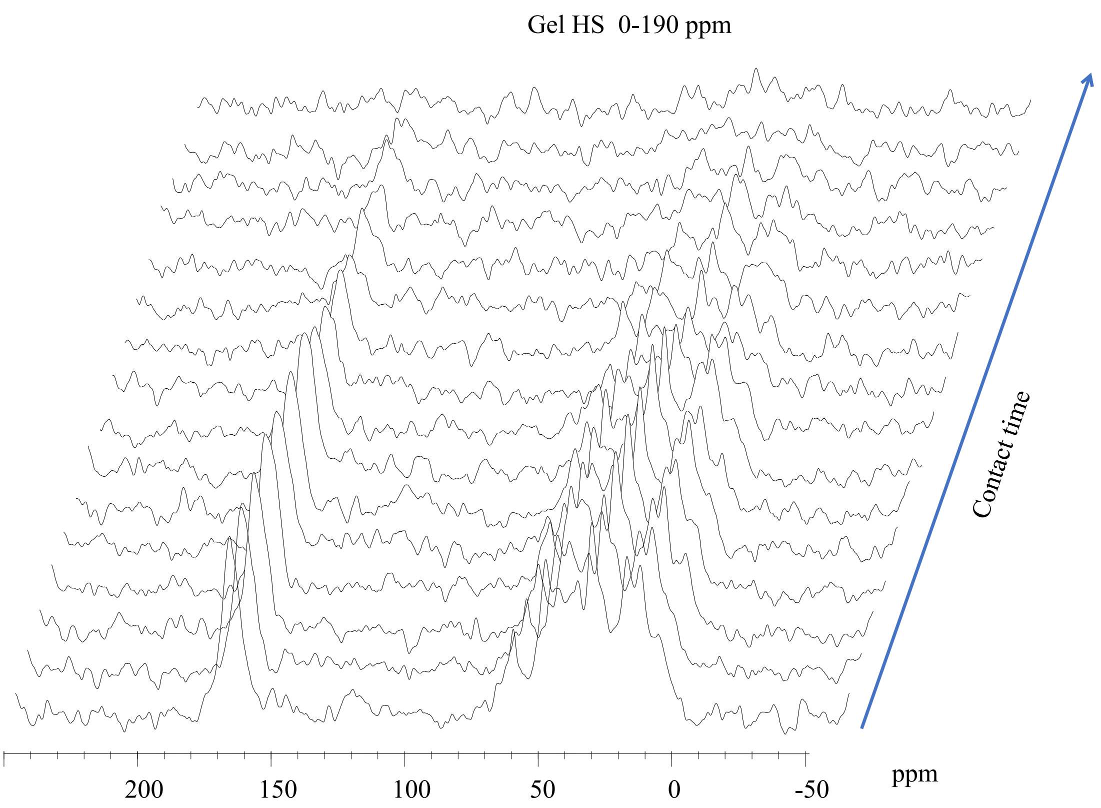

Figure S2b. Representative ^{13}C CPMAS NMR VSL experiments.
